# Modulator of Apoptosis 1: A Highly Regulated RASSF1A-Interacting BH3-Like Protein

**DOI:** 10.1155/2012/536802

**Published:** 2012-06-14

**Authors:** Jennifer Law, Victor C. Yu, Shairaz Baksh

**Affiliations:** ^1^Department of Pediatrics, Faculty of Medicine and Dentistry, University of Alberta, 3055 Katz Group Centre for Pharmacy and Health Research, 113 Street 87 Avenue, Edmonton, AB, Canada T6G 2E1; ^2^Department of Pharmacy, Faculty of Science, National University of Singapore, 18 Science Drive 4, Singapore 117543

## Abstract

Modulator of apoptosis 1 (MOAP-1) is a BH3-like protein that plays key roles in both the intrinsic and extrinsic modes of cell death or apoptosis. MOAP-1 is part of the Ras association domain family 1A (RASSF1A)/MOAP-1 pro-apoptotic extrinsic signaling pathway that regulates apoptosis by utilizing death receptors such as tumor necrosis factor **α** (TNF**α**) or TNF-related apoptosis-inducing ligand (TRAIL) to inhibit abnormal growth. RASSF1A is a bona fide tumor suppressor gene that is epigenetically silenced by promoter-specific methylation in numerous human cancers. MOAP-1 is a downstream effector of RASSF1A that promotes Bax activation and cell death and is highly regulated during apoptosis. We speculate that MOAP-1 and RASSF1A are important elements of an “apoptotic checkpoint” that directly influences the outcome of cell death. The failure to regulate this pro-apoptotic pathway may result in the appearance of cancer and possibly other disorders. Although loss of RASSF1A expression is frequently observed in human cancers, it is currently unknown if MOAP-1 expression may also be affected during carcinogenesis to result in uncontrolled malignant growth. In this article, we will summarize what is known about the biological role(s) of MOAP-1 and how it functions as a downstream effector to RASSF1A.

## 1. Introduction

Cancer is a disease of uncontrolled cell proliferation and is the third leading causing of death worldwide following cardiovascular and infectious diseases [2]. The abnormal proliferation of cells during cancer development results from a multistep process involving the deregulation of genes that promote cell growth (oncogenes) and those that normally function to restrain growth (tumor suppressors). Interestingly, approximately 90% of the genes that are associated with cancer development have now been identified as being tumor suppressors [3]. Moreover, many of these growth inhibitory genes encode proteins that are involved in cell death. RASSF1A has multiple biological functions including the regulation of Bax-mediated cell death [4–6]. MOAP-1, a highly regulated pro-apoptotic protein, serves a critical role during mitochondrial-dependent apoptosis by influencing and sustaining Bax activation [7, 8]. In this review, we will discuss how MOAP-1 is regulated and how it serves as a pivotal RASSF1A effector protein to regulate cell death.

## 2. Apoptosis: A Regulated Biological Process to Modulate Growth

 A well-known mechanism of tumor suppression is the elimination of unwanted cells through a sequence of events known as *apoptosis* [9]. The significance of apoptosis in metazoan biology is highlighted by the number of diseases that are associated with its deregulation [10]. Apoptosis plays a critical role during the development of multicellular organisms and adult tissue homeostasis and is vital to the removal of damaged or dangerous cells. It can be initiated through two main pathways in response to intracellular or extracellular signals of cell death [11]. The intrinsic apoptotic signaling pathway is activated in response to a diverse set of signals originating from within cells due to cellular stresses such as DNA damage, hypoxia, toxins, or starvation [12]. In contrast, the extrinsic pathway of cell death is activated by the binding of death-inducing ligands to death receptors.

Activation of the extrinsic apoptotic signaling pathway occurs through cell surface death receptor/ligand combinations that include TNF-R1/TNF*α*, Fas receptor (R) (CD95/APO-1)/Fas ligand, as well as TRAIL-R (1/2)/TRAIL [13]. Activated death receptors trigger a series of events resulting in the formation of trimeric receptor complexes and the death-inducing signaling complex (DISC) [14]. DISC assembly and subsequent activation of initiator caspases (mainly caspase-8) convey signals to the mitochondria to promote the release of small molecules (such as cytochrome c) from the mitochondrial matrix into the cytosol and the assembly of the apoptosome complex to activate downstream effector caspases (such as caspase-3) [15]. Intrinsic pathway stimulation can also lead to cytochrome c release and activation of effector caspases. Once activated, effector caspases cleave several nuclear proteins [such as lamin B and poly(ADP-ribose) polymerase] and activate specific DNA endonucleases. These events result in many of the biochemical and morphological changes observed during apoptosis, including nuclear and cytoplasmic breakdown.

Mitochondria play an important role in the induction of apoptosis through the release of proteins that promote caspase activation and the breakdown of cellular components [16]. Regulation of the mitochondrial events during apoptosis is controlled by proteins of the B-cell lymphoma-2 (Bcl-2) family and is composed of three different subgroups known as the anti-apoptotic, multidomain pro-apoptotic and BH3-only proteins [12, 17]. The anti-apoptotic and BH3-only proteins are involved in inhibiting or promoting the function of multi-domain pro-apoptotic molecules, respectively. In contrast, it is members of the multidomain subgroup that are directly responsible for the mitochondrial outer membrane permeabilization that occurs during apoptosis [18, 19]. Two members from this group, Bax and Bak, are required for apoptosis to occur [20]. Although the functions of Bax and Bak are closely regulated by its Bcl-2 family members, it is now known that, for at least Bax activation, other proteins may also be involved in its modulation. One of these molecules is the RASSF1A-binding protein, MOAP-1. RASSF1A functions to “open” MOAP-1 to allow for MOAP-1-induced Bax conformational change by exposing the epitope, ^12^GPTSSEQIMKTGA^24^, and allowing for the subsequent insertion of Bax into the mitochondrial membrane. Once inserted, Bax can cooperatively drive cell death in association with Bak [21].

## 3. Ras Association Domain Family

RASSF1A is a bona fide tumor suppressor molecule that serves as the founding member of the RASSF group of proteins [22]. Currently, the RASSF protein family is comprised of ten different members known as RASSF1–10 that each share the presence of a Ras association (RA) domain within its primary amino acid sequence [23–26]. Of this protein family, RASSF1 is the most thoroughly characterized and studied thus far. A loss or decrease in RASSF1A expression is frequently observed in a wide range of human cancers due to epigenetic transcriptional silencing [27–30].

The tumor suppressor functions of RASSF1A include the ability to regulate microtubule dynamics [31–33], mitosis [32, 34–37], and apoptosis [5, 6, 38–41]. Due to the particular focus of this paper, we will only discuss in detail what is known about RASSF1A-dependent cell death involving MOAP-1. It is now known that several pro-apoptotic pathways can be modulated by RASSF1A. One such pathway for the induction of RASSF1A-mediated apoptosis involves protein interactions with the Hippo signaling components, serine/threonine kinases mammalian Ste20-like (MST) 1 and 2 (reviewed separately in this issue). The Hippo pathway is a conserved signaling pathway essential for organ growth regulation in *Drosophila *and vertebrates [42]. Currently, there is evidence to support the role for RASSF1A in modulating the kinase activity of MST1/2 and thus MST1/2-mediated cell death [38, 39]. RASSF1A can also induce apoptosis through an MST2-specific pathway by releasing MST2 from its inhibitor, Raf1, and allowing for large tumor suppressor homology (*Drosophila*) (LATS)1-mediated activation of the transcriptional regulator Yes-associated protein (YAP)1 [41]. In turn, YAP1 can translocate to the nucleus and associate with the p73 transcription factor in order to induce the transcription of pro-apoptotic gene p53-upregulated modulator of apoptosis (*PUMA*) to aid in Hippo-mediated cell death.

A second pathway involves MOAP-1. In response to death receptor signaling involving TNF*α* or TRAIL, RASSF1A can associate with MOAP-1 in order to promote Bax conformational change, translocation and integration into the mitochondrial membrane to perturb mitochondrial permeability [5, 6]. This is followed by the release of cytochrome c to activate downstream caspases and to promote nuclear and cytoplasmic breakdown. Furthermore, we speculate that MOAP-1 may cooperate with RASSF1A to promote tumor suppression. RASSF1A has been extensively reviewed in the literature. In contrast, there are currently no reviews that specifically address what is known about the biology of MOAP-1. Indeed, MOAP-1 remains separate from the canonical group of Bax-regulatory molecules and therefore has not garnered as much attention as the proteins of the Bcl-2 family. In the remainder of this review, we will document what is currently known about MOAP-1 and will discuss evidence providing insight into the complexities of this protein and its biological function(s).

## 4. Modulator of Apoptosis 1: A Brief History

**Figure 1:**
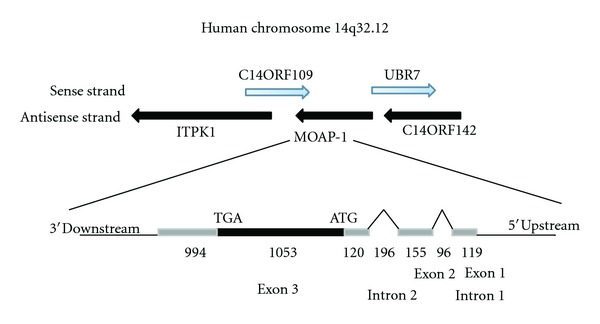
Gene structure of human MOAP-1. The entire protein coding sequence of MOAP-1 is contained within exon 3 and is located on the anti-sense strand of chromosome 14. Genbank accession: NM_022151.4. More information can be found at http://www.ncbi.nlm.nih.gov/gene/64112. Numbers below schematic denote the size of the intron or exon.

**Figure 2:**
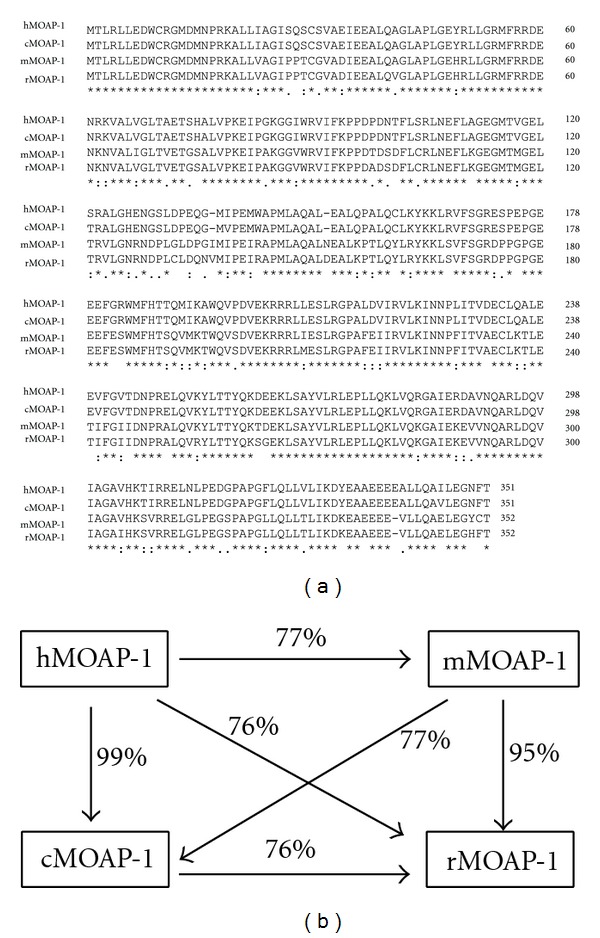
A comparison of MOAP-1 orthologs. (a) Multiple sequence alignments of MOAP-1 orthologs present in human (h), mouse (m), rat (r) and chimpanzee (c). Sequence alignments were performed using ClustalW2. NCBI reference sequences (mRNA and protein): NM_022151.4 and NP_071434.2 (human); NM_022323.7 and NP_071718.1 (mouse); NM_001013101.1 and NP_001013119.1 (rat); XM_510137.3 and XP_510137 (chimpanzee). (b) Percent amino acid identity between MOAP-1 orthologs calculated based on sequence alignments in (2a). Analysis carried out using ClustalW2.

 MOAP-1 was first reported as a mitochondria-enriched 39.5 kDa molecule that was first identified as a novel Bax-associating protein in a yeast two-hybrid screen [7]. Located at genetic locus 14q32 (Figure 1), MOAP-1 is a negatively charged protein that contains 351 amino acid residues in humans and an isoelectric point (pI) of 4.939 at pH 7.0 (Ensembl protein ID: ENST00000298894). *MOAP-1* is highly conserved in chimpanzee (*Pan troglodytes*), rat (*Rattus norvegicus*) and mouse (*Mus musculus*), and its coding sequence is contained within a single exon in both mouse and humans (Figure 2). Since its discovery in 2001, research has established a central role for MOAP-1 in both mitochondrial and death receptor-mediated apoptosis [5, 8]. When overexpressed in mammalian cells, MOAP-1 induces caspase-dependent apoptosis whereas MOAP-1 knockdown cells are resistant to a variety of apoptotic stimuli including staurosporine, serum withdrawal, UV irradiation, TNF*α*, and TRAIL [8]. Altogether, these results demonstrate the importance of MOAP-1 in apoptosis and functions as a key effector of Bax conformational change and activation.

## 5. MOAP-1 Expression in Normal and Cancer Cells

**Figure 3:**
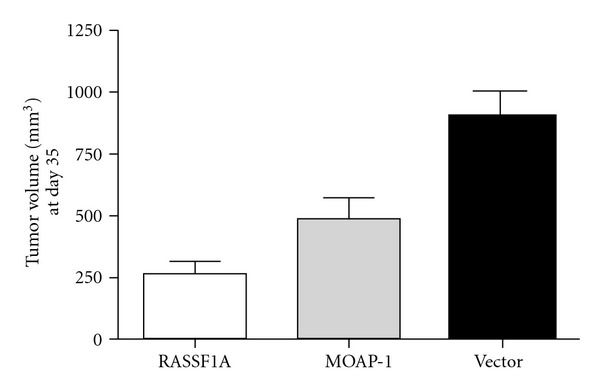
Tumor inhibiting potential of the RASSF1A/MOAP-1 tumor suppressor pathway. A classical xenograft assay was carried out. Male athymic nude mice were injected subcutaneously with 1 × 10^6^ transiently transfected HCT116 cells mixed with matrigel mix into the right and left flank areas. Tumor volumes were measured until day 35 and plotted. *P* values for MOAP-1 versus vector (0.019); RASSF1A versus vector (0.0001); MOAP-1 versus RASSF1A (0.02), *n* = 12–14. Statistical analysis was evaluated by Student's *t*-test (two-tailed). Protein expression at the time of subcutaneous injection was confirmed by immunoblotting (data not shown). Protein expression in HCT116 cells can be detected up to 10 days post-transfection. However, at the end of experiment, we could not detect protein expression of HA-RASSF1A or Myc-MOAP-1 in the resulting tumors. We argue that the growth properties of HCT116 cells containing the indicated expression constructs were programmed within the first 7–10 days and continued on that program even though expression detection of the indicated genes was not possible. Please refer to [1] for more details on this issue.

 MOAP-1 is a ubiquitously expressed protein that is present at moderate levels under normal cellular conditions and is constitutively degraded by the ubiquitin-proteasome system [7, 43]. Given that RASSF1A expression is frequently lost during carcinogenesis and Bax is mutated in a large percentage of gastrointestinal and colorectal cancers, it is plausible that MOAP-1 expression and/or function may also be regulated during cancer development [29, 44, 45]. Indeed, immunohistochemical analysis of MOAP-1 performed over a wide range of human cancer tissues demonstrates either a negative or a weak staining pattern for this protein (Table 1 and please see site http://www.proteinatlas.org/search/moap1 under “moap1 or pnma4” for immunohistochemical pictures of MOAP-1 staining in numerous cancer cells). In support of this immunohistochemical data, we have also found a loss or reduction of MOAP-1 expression in an extensive panel of cancer cell lines ranging from breast, brain, lung, skin and blood cancers [Law et al., unpublished observations]. Furthermore, in a classical xenograft assay, both RASSF1A and MOAP-1 can suppress tumor formation in HCT116 colon cancer cells suggesting tumor suppressor function (Figure 3) and functional importance for both genes in growth inhibition in normal cells.

Currently, the mechanism responsible for the loss of MOAP-1 expression in cancer cells remains unknown. It is possible that expression changes in MOAP-1 may arise by promoter specific epigenetic methylation, by miRNA/siRNA regulation of the mRNA, and/or by alterations in MOAP-1 protein stability due to ubiquitin-directed proteolysis. The *MOAP-1* promoter displays 17 potential CpG islands that may be epigenetically modified to result in loss of gene expression, as suggested using MethPrimer online software [46]. To date, no miRNA or siRNA has been identified for MOAP-1 although we suspect that specific miRNA(s) may exist to reduce or shut down MOAP-1 expression. The last potential mechanism regulating MOAP-1 expression is posttranslational modification by ubiquitination and degradation by the proteasomal degradation machinery [43]. Future investigations will be required in order to understand the ubiquitination of MOAP-1 and the biological outcome of these ubiquitination events.

**Figure 4:**
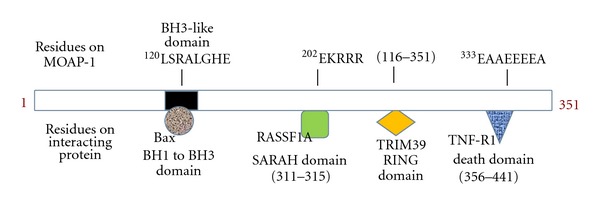
A schematic of MOAP-1 with indicated areas of contact with other proteins documented above schematic. Residues empirically determined to be required for protein interactions are indicated below each region.

Like most disease-associated genes, polymorphisms may exist to result in the loss of the encoded protein function. Single nucleotide polymorphisms (SNPs) of MOAP-1 have been documented in two databases suggesting disease-associated changes [47, 48]. Although the population distribution has not been determined as of yet, two somatically derived SNPs (resulting in a predicted amino acid change) have been observed in melanoma patients—a proline to serine change at amino acid 79 (P79S with a nucleotide change of CCT → TCT) and an alanine to aspartic acid change at position 335 (A335D with a nucleotide change of GCT → GAT) [49]. Interestingly, the P79S polymorphism may suggest the creation of a potentially novel serine phosphorylation site to affect the cell death properties of MOAP-1, whereas A335D amino acid change would affect the TNF-R1-binding site on MOAP-1 (please see Figure 4). Further verification of these SNPs is warranted with respect to penetrance within the normal and disease groups, origin of these potential polymorphic changes, and their biological significance. Regardless of how MOAP-1 may lose expression and/or function, we speculate that the combined loss of both MOAP-1 and RASSF1A expression may be a common event occurring during carcinogenesis to result in the functional loss of the MOAP-1/RASSF1A cell death pathway and enhanced proliferation of malignant cells. Furthermore, the absence of MOAP-1 in cancer cells would also impact to some extent on the intrinsic apoptotic pathway(s) where MOAP-1 has been shown to play a role [8] and which is also the target of many chemotherapeutic drugs. Future investigations will be required in order to determine the cause(s) underlying MOAP-1 expression changes in human cancer.

Evidence from the literature indicates downregulation of *MOAP-1* expression in macrophage cells upon overexpression of the transcription factor MafB [50]. Upregulation of MafB is commonly observed in alveolar macrophages that have been exposed to cigarette smoke, and, incidentally, these cells also display increased viability [50, 51]. It has been proposed that MafB may promote macrophage survival through inhibition of apoptosis, which may be achieved through downregulation of pro-apoptotic molecules such as MOAP-1 [50]. In addition, analysis of the promoter region of MOAP-1 for transcription factor binding sites identified several interesting sites for NF*κ*B (CCCTGGTCCC CAAGGAAATA CCT GCAAAAG) and c-Rel (ATCGGAATGA CCCTCTCGGC) and three sites for STAT1 (CTTGCTCCCT TAGGGGAACA) using the online, publicly available Transcription Factor Search (TFSEARCH) software. It remains to be determined if these are functional transcription factor binding sites but does provide hints to the complexity of MOAP-1 expression and reaffirms its importance in both cell death and growth control.

## 6. Interaction of MOAP-1 with Bcl-2 Family Members

As a pro-apoptotic molecule, MOAP-1 selectively interacts with members of the Bcl-2 protein family. In particular, its association with Bax requires the presence of a Bcl-2 homology 3 (BH3)-like domain within amino acids 120–127 and the same domain is also essential for mediating apoptosis [7]. Interestingly, the association of MOAP-1 to Bax requires all three BH (BH1, BH2, BH3) domains of the latter protein and is thus in contrast to other known Bax-associating partners (Figure 4). Additionally, it is speculated that MOAP-1 may associate at the hydrophobic cleft of Bax since critical point mutations in any of the three BH domains in Bax result in a loss of MOAP-1 association. The interaction between MOAP-1 and Bax occurs upon induction of apoptosis in response to activators of both the intrinsic and extrinsic cell death pathways and facilitates the release of cytochrome c from the mitochondria [8].

In addition to Bax, MOAP-1 also associates with the prosurvival anti-apoptotic proteins Bcl-2 and Bcl-X_L_ but not additional Bcl-2 family members Bid, BimL, Bak, Bad or Bcl-w under the same experimental conditions [7]. Evidence suggests that its interactions with Bcl-2 and Bcl-X_L_ may function to restrain the pro-apoptotic activity of MOAP-1 since overexpression of Bcl-X_L_ is sufficient to block MOAP-1-mediated cell death. Therefore, it appears that MOAP-1 may function similar to the canonical BH3-only proteins of the Bcl-2 family that are known to promote Bax activation and which are also inhibited by its anti-apoptotic family members.

## 7. Cooperation of MOAP-1 with RASSF1A in Death Receptor-Mediated Apoptosis

**Figure 5:**
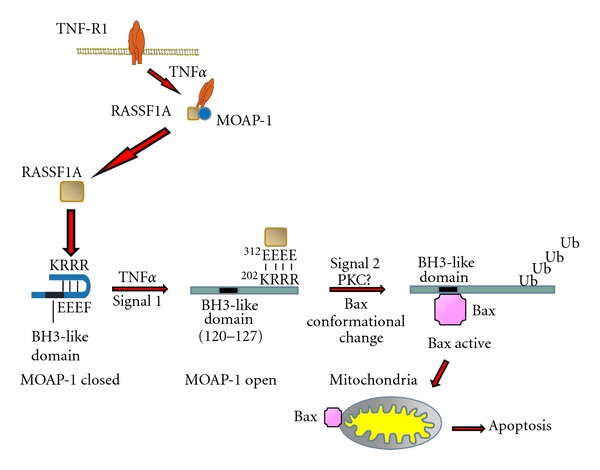
MOAP-1 cooperates with RASSF1A during death receptor-dependent apoptosis and promotes Bax activation. In response to death receptor stimulation, MOAP-1 is first recruited to the receptor and then followed by RASSF1A association at the MOAP-1/receptor complex. The association of MOAP-1 to RASSF1A promotes a conformational change in MOAP-1 that exposes its BH3-like domain required for Bax association. The subsequent interaction between MOAP-1 and Bax induces a conformational change in Bax that enables its translocation, and insertion into the mitochondrial outer membrane resulting in the release of cytochrome c and other apoptogenic factors, leading to apoptosis.

MOAP-1 is required for execution of both the intrinsic and extrinsic pathways of apoptosis where it is required for Bax conformational change and translocation from the cytosol to the mitochondria prior to the release of apoptogenic factors [5, 8]. Although the mechanistic details of its role in the intrinsic pathway are currently unknown, the death receptor-dependent pathway involving MOAP-1 has been delineated to a great extent [5, 6] (Figure 5).

Under nonstimulated conditions, MOAP-1 is normally held in a “closed” conformation through an intraelectrostatic interaction involving regions ^178^EEEF and ^202^KRRR [6]. However, stimulation of cells with TNF*α* or TRAIL results in the recruitment of MOAP-1 to the receptor via a basic sequence (^336^EEEEA) at its C-terminal end (Figures 4 and 5). Prior to death receptor association, RASSF1A is released from association with 14-3-3 and loses its ability to homodimerize [52]. Upon binding to the receptor through its N-terminal cysteine-rich (C1) domain, RASSF1A induces a conformational change in MOAP-1 to a more “open” state (Figure 5, Signal 1, TNF*α*) that exposes its BH3-like domain and allows it to bind and promote the activation of Bax [6].

The association of MOAP-1 with RASSF1A involves the sequence ^202^KRRR in the former protein and ^312^EEEE in the latter. Although activated K-Ras has been reported to be required for stabilization of the MOAP-1/RASSF1A protein complex [53], we are is able to consistently detect robust associations between MOAP-1 and RASSF1A in experiments that do not require the presence of overexpressed active K-Ras [5, 6]. Therefore, we are currently unable to explain or support the results of Vos and colleagues. Nonetheless, MOAP-1-induced Bax conformational change enables Bax to translocate from the cytosol to the mitochondria where it can insert into the mitochondrial membrane and promote the release of cytochrome c as well as other apoptosis-inducing factors, resulting in cell death. Therefore, MOAP-1 functions alongside RASSF1A as a key component linking death receptor signaling to Bax activation and mitochondria-associated cell death. The MOAP-1/RASSF1A pathway exists as a separate, parallel signaling cascade that links the extrinsic and intrinsic pathways of apoptosis independent of tBid and caspase 8 [5].

In addition to RASSF1A, MOAP-1 has also demonstrated the ability to associate with a second RASSF family member, RASSF6 [54]. The interaction between RASSF6 and MOAP-1 is enhanced by the presence of activated K-Ras, and, furthermore, RASSF6 is also able to promote apoptosis. Therefore, it has been speculated that Ras may activate the pro-apoptotic function of RASSF6 and that RASSF6 may cooperate with MOAP-1 in a pathway similar to RASSF1A in order to induce cell death. However, this hypothesis still needs to be verified.

## 8. Regulation of MOAP-1 Stability by Apoptotic Signals

 Under nonstimulated conditions, MOAP-1 is constitutively degraded by the ubiquitin-proteasome system and is normally a short-lived protein with a half-life of approximately 25 minutes [43]. However, evidence suggests that targeting of MOAP-1 to the proteasome may involve an unconventional mechanism given that no specific lysine residue can be identified as the site of polyubiquitination [43]. Indeed, mutation of any individual lysine residue or combination of residues fails to abolish MOAP-1 ubiquitination. Thus, the process involved in controlling MOAP-1 turnover remains to be determined.

In addition to regulation of basal MOAP-1 expression, MOAP-1 is also rapidly upregulated in response to multiple apoptotic stimuli including serum withdrawal, etoposide, TRAIL, and the endoplasmic reticulum stress inducer thapsigargin [43]. The increase in MOAP-1 protein arises through inhibition of its polyubiquitination and subsequent proteasomal degradation. Research findings demonstrate that elevation of MOAP-1 levels occurs prior to cell commitment to apoptosis and that the stabilization of MOAP-1 helps to sensitize cells to apoptosis by increasing the levels of activated Bax.

 Intriguingly, stabilization of MOAP-1 in response to apoptosis employs the RING domain protein tripartite motif containing 39 (TRIM39) [55]. TRIM39 has not yet been functionally characterized but belongs to the tripartite motif (TRIM) family of proteins that are commonly involved in innate immunity [56] and contains three zinc-binding domains including a RING, B box, and coiled-coil region. Although a large number of proteins that contain RING domains also function as E3 ligases [57], TRIM39 associates with MOAP-1 in a manner that promotes its stabilization rather than its polyubiquitination [55]. TRIM39 also sensitizes cells to apoptosis by inhibiting MOAP-1 ubiquitination (through an unknown mechanism) and thus allows for the accumulation of MOAP-1 that can then can activate Bax. Furthermore, it was observed that both TRIM39 and MOAP-1 influence each other's localization to the mitochondria when overexpressed in HEK293 cells [55]. The upregulation of MOAP-1 protein levels can also occur in response to chemical toxins and clinical drugs reaffirming our speculation that MOAP-1 in cancer cells may be important for patient response to certain chemotherapeutic treatments [58]. Incubation of chronic lymphocytic leukemia cells with the apoptosis-inducing compound 5-aminoimidazole-4-carboxamideriboside or acadesine (AICAR) has been shown to result in a significant increase in *MOAP-1* expression [58]. Although the pathway through which AICAR induces cell death remains unknown, it is achieved through a mechanism that is independent of both AMPK and p53. In a second example, the addition of the novel immunosuppressant 2-amino-2[2-(4-octylphenyl) ethyl]-1,3-propane-diol hydrochloride (FTY720) to Jurkat cells results in a greater than tenfold upregulation of MOAP-1 mRNA levels [59]. It is believed that the potent immunosuppressive function of FTY720 may be attributed to its ability to induce lymphocyte apoptosis [60]. However, FTY720 has also been shown to induce apoptosis in a variety of different cancer cell types and to prevent breast cancer metastasis in mouse models [61–64]. Thus, it is plausible that the immunosuppressive and/or antitumorigenic effects of FTY720 may be partially mediated by MOAP-1.

 We have evidence for a nondegradative ubiquitination of MOAP-1. This post-translational modification proceeds through a mechanism that is responsive to death receptor stimulation and a novel protein kinase C (PKC) dependent event [Law et al, unpublished observations] that may allow MOAP-1 to associate with and promote Bax activation (Figure 5, Signal 2). Interestingly, MOAP-1 has two potential binding sites for TRAF2, an E3-ubiquitin ligase important for TNF-R1-dependent signaling. These sites are at ^178^EPGEEFGRW AND ^331^DYEAAEEEAL with the underlined residues forming the core of the TRAF2 association site [64]. The first potential site is part of the intraelectrostatic pair that overlaps with the BH3-domain of MOAP-1. We are currently investigating the possible involvement of TRAF2 in MOAP-1 ubiquitination and the functional importance of several potential lysine residues for ubiquitin-dependent modification. We speculate that the ubiquitination of MOAP-1 may influence MOAP-1-mediated growth suppression and/or MOAP-1-directed apoptosis. This form of MOAP-1 ubiquitination adds to the complexity of MOAP-1 stability by a degradative-dependent ubiquitination to modulate the biological functions of MOAP-1.

## 9. MOAP-1: A Paraneoplastic Antigen

 In addition to its role as a pro-apoptotic molecule, MOAP-1 is also the fourth member of the paraneoplastic Ma antigen (PNMA) family and is consequently also known as PNMA4. Paraneoplastic antigens (also termed “onconeural antigens”) are proteins that are restricted in expression to immune-privileged sites within the body (such as the brain) and are therefore recognized as foreign molecules by the immune system when aberrantly expressed at other sites [65, 66]. Remarkably, these foreign proteins are expressed by systemic tumors in a subset of cancer patients which subsequently trigger an immune-mediated antitumor response. In some patients, this immune response is not only directed against the tumor itself but also towards the sites within the body that ordinarily express the protein. In the case of the brain, this immune response results in neuronal degeneration and the development of an autoimmune neurologic disease known as a paraneoplastic neurological disorder (PND).

The PNMA family consists of six members (PNMAs 1–6) that, with the exceptions of PNMAs 4, 5, and 6, were originally identified through screening of complementary DNA libraries using antibody-containing sera from patients with PNDs [67]. Although MOAP-1/PNMA4 is ubiquitously expressed with higher levels in the heart and brain [7], each of the other family members are more restricted in expression [67–71]. The detection of antibodies to PNMAs 1–3 in PND patients is associated with disorders affecting the limbic system, brain, stem and cerebellum but is not indicative of any particular cancer type [68–70, 72]. In contrast, MOAP-1 has a well-established role in apoptosis and—similar to PNMA5 and PNMA6—is not associated with the development of PNDs to date. MOAP-1 displays the greatest amino acid sequence homology with PNMA1 (58%) which functions as a neuronal-specific pro-apoptotic molecule [73]. PNMA1 contains both a BH3-like domain and a conserved RASSF1A association site similar to that found on MOAP-1 (Figure 4). However, PNMA1 does not associate with either Bax or RASSF1A [73], and, therefore, although unknown, the mechanism by which it induces cell death presumably differs from MOAP-1. It remains to be determined how, and if, MOAP-1 may impinge on the pathogenesis of paraneoplastic syndromes.

## 10. Concluding Remarks

MOAP-1 is a highly regulated pro-apoptotic molecule that demonstrates multiple potential properties of a candidate tumor suppressor protein. Given that MOAP-1 regulates RASSF1A pro-apoptotic function and RASSF1A is also epigenetically silenced in a large number of human cancers, it is possible that the combined loss of MOAP-1 and RASSF1A during carcinogenesis may result in the inhibition of extrinsically activated cell death signaling pathways in cancer cells. RASSF1A has now been demonstrated to influence several other biological processes such as cell cycle, microtubule dynamics, and cell migration. Therefore, it will be interesting to explore which of these biological processes MOAP-1 may also be involved in and that may be important for it to behave as a potential tumor suppressor protein.

## Figures and Tables

**Table 1:** Summary of MOAP-1 staining patterns in human malignant tissues. Data source was The Human Protein Atlas (http://www.proteinatlas.org/search/moap1). Antibody used for all MOAP-1 immunohistochemistry: Sigma-Aldrich HPA000939.

Cancer tissue type	MOAP-1 staining pattern
Colorectal cancer	Weak
Breast cancer	Negative
Prostate cancer	Negative
Ovarian cancer	Negative
Cervical cancer	Negative
Endometrial cancer	Negative
Malignant carcinoid	Negative
Head and neck cancer	Negative
Thyroid cancer	Negative
Malignant glioma	Weak
Malignant lymphoma	Negative
Lung cancer	Weak
Malignant melanoma	Negative
Skin cancer	Negative
Testis cancer	Moderate
Urothelial cancer	Negative
Renal cancer	Negative
Stomach cancer	Weak
Pancreatic cancer	Negative
Liver cancer	Negative

## Abbreviations

AICAR: 5-Aminoimidazole-4-carboxamideribosideor acadesine
Bcl-2: B-cell lymphoma-2
BH3: Bcl-2 homology 3
FTY720: 2-Amino-2[2-(4-octylphenyl) ethyl]-1,3-propane-diol hydrochloride
LATS1: Large tumor suppressor, homolog 1
MOAP-1: Modulator of apoptosis
MST: Mammalian sterile 20-like
pI: Isoelectric point
PND: Paraneoplastic neurological disorder
PNMA: Paraneoplastic Ma antigen
RAS: Rat sarcoma
RASSF: Ras association domain family
RA: Ras association domain
SNP: Single nucleotide polymorphism
TNF*α*: Tumor necrosis factor *α*
TNF-R1: Tumor necrosis factor receptor 1
TRAIL: TNF-related apoptosis-inducing ligand
TRIM39: Tripartite motif containing 39
YAP1: Yes-associated protein 1
